# Fast and slow myosins as markers of muscle injury

**DOI:** 10.1136/bjsm.2007.037945

**Published:** 2007-12-07

**Authors:** M Guerrero, M Guiu-Comadevall, J A Cadefau, J Parra, R Balius, A Estruch, G Rodas, J L Bedini, R Cussó

**Affiliations:** 1 University of Barcelona, Barcelona, Spain; 2 Centre d’Estudis d’Alt Rendiment Esportiu (CEARE), Barcelona, Spain; 3 FC Barcelona (FCB), Barcelona, Spain; 4 Hospital Clínic i Provincial de Barcelona, Barcelona, Spain

## Abstract

**Objective:**

The diagnosis of muscular lesions suffered by athletes is usually made by clinical criteria combined with imaging of the lesion (ultrasonography and/or magnetic resonance) and blood tests to detect the presence of non-specific muscle markers. This study was undertaken to evaluate injury to fast and slow-twitch fibres using specific muscle markers for these fibres.

**Methods:**

Blood samples were obtained from 51 non-sports people and 38 sportsmen with skeletal muscle injury. Western blood analysis was performed to determine fast and slow myosin and creatine kinase (CK) levels. Skeletal muscle damage was diagnosed by physical examination, ultrasonography and magnetic resonance and biochemical markers.

**Results:**

The imaging tests were found to be excellent for detecting and confirming grade II and III lesions. However, grade I lesions were often unconfirmed by these techniques. Grade I lesions have higher levels of fast myosin than slow myosin with a very small increase in CK levels. Grade II and III lesions have high values of both fast and slow myosin.

**Conclusions:**

The evaluation of fast and slow myosin in the blood 48 h after the lesion occurs is a useful aid for the detection of type I lesions in particular, since fast myosin is an exclusive skeletal muscle marker. The correct diagnosis of grade I lesions can prevent progression of the injury in athletes undergoing continual training sessions and competitions, thus aiding sports physicians in their decision making.

Muscle is sensitive to the protocols of contraction and work to which it is submitted, since its structure is designed to support these protocols and to adapt to new situations of force. However, if the integrity is affected to a greater or lesser extent by overload, tears occur which are known as muscle lesions. These lesions can result in incapacity to continue exertion of the force.

Extenuating unaccustomed exercise and high-force eccentric action leads to skeletal muscle damage with changes in muscle structure and function. It induces damage to muscle fibre membranes,^1^ ^2^ myofibrillar disruption^3^ and sarcoplasmic reticulum vacuolisation.^4^ Such exercise-induced muscle damage activates a cascade of reactions that result in activated skeletal muscle protein metabolism. The protease calpain is activated immediately after exercise. Calpain initiates the metabolic turnover of myofibrillar proteins by releasing them from their filamentous structure.^5^ Although calpain does not degrade actin and myosin, it contributes to their release.^6^ This allows the detection of such proteins in peripheral blood after cleavage, using specific assays such as troponin I (TnI) and myosin heavy chains (MHC).^7^ ^8^ Sorichter *et al*^9^ described the features of an ideal marker of skeletal muscle fibre injury. One of these was that the marker should be absolutely muscle fibre-specific to allow reliable diagnosis of skeletal fibre type injury. None of the markers analysed by these authors is muscle type-specific.

The markers normally used include creatine kinase (CK), heart-fatty acid binding protein, myoglobin, TnI and α-actin.^10^ However, in addition to not being totally specific for skeletal muscle, they reach a maximum value before 10 h have elapsed after the origin of the lesion and decrease to a considerable extent within 24 h. Most lesions occur during holidays, so it is very easy for these 10–12 critical hours to pass before the patient is examined. New lesions are often not accompanied by pain, but a day later the markers of low molecular weight have degraded with no trace left in the serum. The troponins are proteins that are very specific in terms of fibre type; they are of low molecular weight but are susceptible to being rapidly proteolysed, which may explain their very short half-life in blood.^11^

Skeletal muscle is a heterogeneous tissue composed of fibre types I and II, the proportion of which varies with the type of muscle and even within the different regions of a particular type of muscle.^12^ Some of the contractile proteins have different isoforms depending on the type of fibre. One of these is myosin, which has different heavy and light isoforms depending on whether the fibre type is fast or slow.^13^

Myosin presents an ideal profile as a parameter to study and is directly assignable to the grade of the lesion since, because of its high molecular weight, its appearance in blood can only be explained by a fibre lesion. Fast myosin is characteristic of fast skeletal muscle only, while slow myosin is common to skeletal and cardiac muscle. The level of slow myosin in the blood has been measured by Schiaffino and Reggiani^14^ and reaches a maximum 48 and 72 h after the lesion.

The aim of this study is to evaluate muscle lesions using as markers fast and slow myosins present in the serum of athletes 48 h after suffering a lesion. The efficiency of these markers is compared with the detection of the lesion by ultrasonography (US), magnetic resonance (MR) and other traditional serum markers.

## METHODS

### Materials

The materials used in the study were monoclonal anti-myosin (skeletal, fast) clone My-32 (Sigma, Madrid, Spain), monoclonal anti-myosin (skeletal, slow) clone NOQ7.5.4D (Sigma), agarose (Sigma), protein A (Sigma) and loading buffer Nupage LDS sample buffer (Novex, California, USA). 

### Subjects

Thirty-six sportsmen aged 18–25 years (athletes, jockeys, tennis players, football players, basketball players and pentathletes) who had suffered some pain and/or injury were studied. The control group comprised 51 non-sportsmen aged 18–55 years.

Muscle injuries were classified into three categories according to clinical findings: grade I (delayed onset muscle soreness and elongation, very small muscle tear); grade II (fibrillar disruption, moderate muscle tear); grade III (fibre disruption, evident muscle tear).  Two ml of blood were obtained from the 51 controls and from the athletes 48 h after they had suffered the muscle problem. Serum was used for measurement of myosin levels.

### Treatment of the sample

The blood samples were obtained in a Vacutainer tube and centrifuged at 2000*g* at 4°C for 10 min. The serum could be kept at −80°C without any loss of myosin for 15 days. Serum protein was determined by the Bradford method.^15^ Myosin is present as a blood marker at a very low concentration compared with other serum proteins. It was therefore concentrated from the serum sample by immunoprecipitation with A protein linked to agarose-antibody. The pellet was resuspended with 15 µl loading buffer (60 mM HCl-Tris, 10% glycerol, 2% SDS, 5% β-mercaptoethanol, 0.025% bromophenol blue, pH 6.8), centrifuged and warmed for 10 min to 70°C. The sample (about 25 µl) was prepared to be carried out on Nupage Novex 3–8% Tris-acetate gels and immunodetection. One or two fast myosin and slow myosin standards were loaded in every gel. Electrophoresis was run in X Cell SureLock Electrophoresis Cell (Invitrogen) at 150 V for 1 h at room temperature. The gel was cut into strips of 7×1.2 cm. The gel for immunoblot analyses was transferred to a PVDF sequencing membrane (Immobilon PSQ Millipore) at 33 V for 70 min at room temperature.

The blots were treated with SuperBlocking buffer in PBS (Pierce) at 0.01% to Tween 20 for 1.5 h at room temperature. One gel was reacted with 1:90 000 anti-myosin fast monoclonal antibody and the other with 1:150 000 anti-myosin slow monoclonal antibody. Membranes were washed with 0.01% Tween 20 at PBS six times for 5 min and incubated with horseradish peroxidase conjugated rabbit anti-mouse IgG (1:90 000) for 1 h at room temperature, followed by additional washes (six times for 5 min in 0.01% Tween 20 at PBS). Each strip was mixed with 80 µl of a mix (1:1) of Ultra Supersignal for 5 min. Proteins were visualised by enhanced chemiluminescence (SuperSignal West Dura Trial Kit, Pierce, Rockford, Illinois, USA). After drying, the strips were printed on the Hyperfilm TM ECL for 10 min. This process was prepared with two samples, one for detecting fast myosin and the other for slow myosin. 

The films were scanned with a Hewlett Packard Scanjet 5200C and quantification was done with Quantity One 1-D (BioRad, Hercules, California, USA).

### Fast and slow myosin standards

The protein standards were prepared from fast and slow rabbit muscle (tibialis anterior for fast fibres and soleus for slow fibres).  Purified myofibrils were obtained according to the method described by Hasten *et al*.^16^ After carrying out this process, myofibril suspensions of about 1 mg/ml of protein were obtained. The suspension was denatured with the loading buffer 1:1 v/v for 2 min at 100°C. 70 µg of protein was applied to an acrylamide gel (7.5% T, 2.5% C) to separate the proteins. The electrophoresis was run for 75 min at 150 V. The presence of myosin was localised by staining a gel with Coomassie Blue G-250 R-250 (BioRad). The bands from an undyed gel were cut and electroluted with a commercial electroluter (BioRad Model 422) during 60 mA, 5 h with the electrolution buffer (25 mM HCl-tris, 192 mM glycine, 0.1% SDS, pH 6.8). We obtained 0.93 μg/μl fast myosin and 1.55 μg/μl of slow myosin. The myosins were diluted with 10 mM HCl-Tris, 300 mM NaCl, 2 mM EDTA at pH 6.8. Standards were kept in fractions of 50 µl at −30°C.

### Measurement of enzymatic activities

CK was determined by a Technicon DAX System autoanalyser according to the method of Szasz *et al*.^17^

### Imaging evaluation

Echography was carried out at the Centre d’Estudis d’Alt Rendiment Esportiu (CEARE) and in the ultrasonographic department of the FIATC Clinic using Toshiba Medical System ultrasonography equipment with a multifrequency probe (Just-Vision in CEARE, PowerVision in FIATC).  Magnetic resonance studies were done at the department of magnetic resonance of the Corachán Clinic using a Siemens Symphony device (1.5 TESS).

Both types of soundings gave results which increased in direct proportion with the grade of the lesion. In grade I lesions US usually shows the lesion (haematic suffusion and defect of some fibres) 2–3 days after the accident while MR shows muscular oedema from the time the lesion occurs. In grade II lesions both US and MR show oedema and fibrillar defects, and grade III lesions show a greater defect associated with haematoma and muscle. Both US and MR show the evolution of the lesion, observing how the oedema disappears and fibrillar repair occurs.

### Statistical analysis

The levels of the each marker in controls and subjects with different grades of injury were compared by analysis of variance (ANOVA); p values of <0.05 were considered significant.

## RESULTS

### MR images

**Figure 1:**
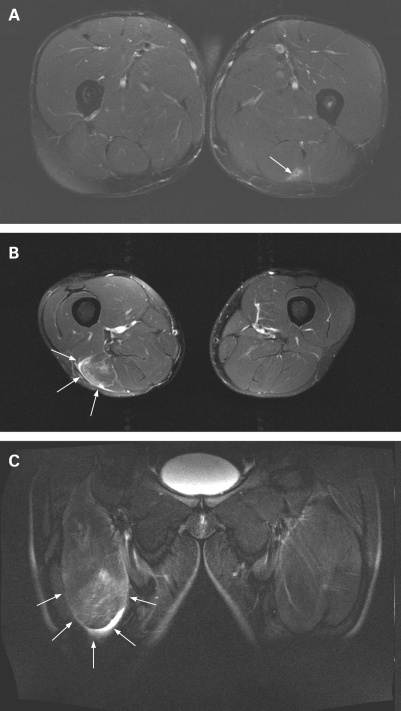
Magnetic resonance images of different grades of muscle lesion: (A) grade I lesion; (B) grade II lesion; (C) grade III lesion.

Figure 1 shows MR images of three different grades of muscle lesion. Figure 1A is an axial view of a grade I lesion in the upper part of the posterior side of the right thigh showing a zone of muscle oedema (signal increase) transduced by a recent tear of the biceps femoris (arrows). Figure 1B is an axial view of a grade II lesion in the medial-distal part of the posterior side of the left thigh showing an area of oedema (signal increase) and fibrillar defect transduced by a recent lesion in the long head of the biceps femoris (arrows). In the coronal view of a grade III lesion of the distal part of the anterior side of the thigh shown in fig 1C, a large area of oedema (signal increase) and extensive fibrillar defect of the rectus femoris can be seen in the distal part (arrows).

### US images

**Figure 2:**
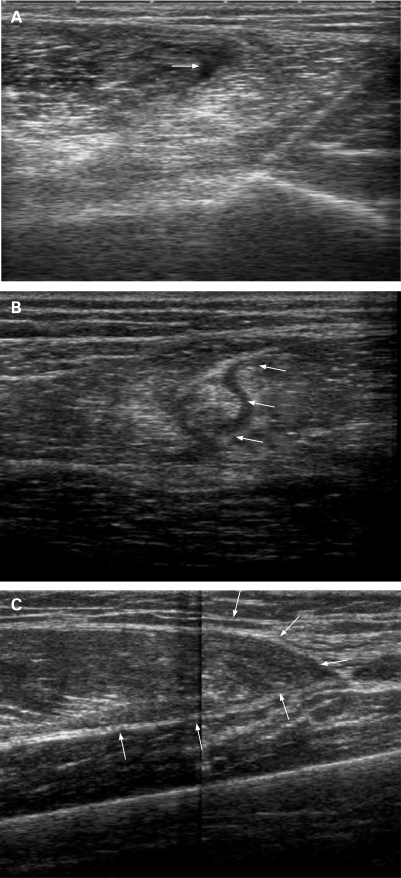
Ultrasonographic appearance of different grades of muscle lesion: (A) grade I lesion; (B) grade II lesion; (C) grade III lesion.

Figure 2 shows the US appearance of the lesions shown in fig 1. Figure 2A is a transverse section of a grade I lesion showing the area of fibrillar defect located between the biceps femoris and the semitendinous. The transverse section of a grade II lesion shown in fig 2B shows the most extensive area of the fibrillar defect and haematic suffusion in the long head of the biceps femoris. The US appearance of a longitudinal section of a grade III lesion in fig 2C shows complete muscle defect of the rectus femoris (arrows).

### Clinical diagnosis, marker enzymes and myosin in normal and injured muscles

Table 1 shows normal serum values of the control group and of the athletes with different grades of muscle lesions. Our results indicate that, in the normal state, the concentration of fast and slow myosins in blood did not exceed 1000 μg/ml fast myosin and 2000 μg/ml slow myosin, showing a fast/slow ratio of 0.3. The patients diagnosed with grade I lesions, which were not imaged by US or MR, had high levels of fast myosin (greater than slow myosin), showing a fast/slow ratio of >2. CK levels were almost within the limits of normality. In grade II and III lesions, which were diagnosed by US and MR, an increase in both fast and slow myosins was observed with a fast/slow ratio near to 1. The concentration of slow myosin compared with fast myosin increased in direct proportion to the severity of the lesion. CK levels also increased in the same direction as the severity of the muscle lesion, which suggests that it is a particularly good marker for grade II and III lesions.

**Table 1:** Comparison of results obtained by different methods for diagnosis of muscle injuries

Diagnosis	No of samples	US	MR	CK (U/l)	Fast myosin (μg/l)	Slow myocin (μg/l)	Fast/slow ratio
Normal	51	–	–	102 (8)	625 (62)	1535 (166)	0.3
Grade I	12	(−) o (+)	–	202 (22)	2880 (159)	1281 (197)	2.2
Grade II	16	++	++	482 (47)	3432 (402)	3722 (700)	0.9
Grade III	10	+++	+++	739 (245)	8055 (2200)	6518 (124)	1.2

Data are expressed as mean (SE).

US, ultrasonography; MR, magnetic resonance; CK, creatine kinase.

Statistical ANOVA was found to be very significant for every parameter (p<0.001).

## DISCUSSION

Human muscles are made up of a mixture of slow and fast fibres with approximately 50% of each. This is different from animals, some of which have 90% of fast fibres and some up to 90% of slow fibres. The vastus lateralis of young Caucasian athletes aged 15–18 years has 36.5% slow-type fibres and 63.5% fast-type fibres and, of these, 52.3% are type IIa, 8.1% type IIb and 3.1% type IIc.^18^ The existence of mixed muscles in humans means that lesions allow the entrance of both slow and fast myosins into the blood. However, due to the fact that resistance to lesions and to fatigue in the two types of fibres is not the same, the level of slow or fast myosin heavy chain (MHC) in the blood depends on the type of fibre damaged. In general, fast fibres are more easily fatigued and are more sensitive to the lesion. We can therefore expect that fast fibres release fast MHC before slow fibres in the face of less intense exercise. Slow MHCs will flow under more fatiguing conditions and their presence in the blood will probably indicate a more important lesion.

On the other hand, the presence of fast MHC in blood signals the fact that only skeletal muscle is affected and thus constitutes an absolutely specific marker. The presence of slow MHC could indicate the presence of a lesion in the skeletal and/or heart muscle. However, given the fact that the subjects of this study were athletes in whom a cardiac lesion is ruled out, the detection of slow MHC in blood would act as a marker of a slow fibre lesion with the consequences that this information would be able to contribute.

We have developed a method to detect myosins in blood based on specific recognition by fast and slow myosin antibodies. We studied a group of athletes involved in different sports who presented with muscle pain. The patients underwent a medical examination, US, MR and blood tests including measurement of CK levels (used as a usual muscle marker) and slow and fast myosins. We also studied 51 people who did not practise any sport either sporadically or for pleasure and who were categorised as normal.

Our results indicate that, 48 h after the onset of the muscle problem, CK activity showed a small increase in grade I lesions. Only fast myosin is a marker with very high values, suggesting that grade I lesions are mainly produced in type II fibres. The results of US and MR in grade I lesions are confusing in some cases, with negative or positive results that fail to assure total recognition of the lesion. US and MR in grade II and grade III lesions were highly effective in detecting the lesions.

In grade II lesions measurement of the serum markers showed an increase in CK activity, with the greatest increases being those of both types of myosin, reaching up to 10 times the normal value in some cases. Grade III lesions were well detected by CK activity and also by both types of myosin.

We conclude that the use of fast myosin provides a highly sensitive marker for grade I lesions, at least equal to MR and better than US and clinical diagnosis. Fast myosin is a totally specific marker for skeletal muscle, a property not shared by any of the markers currently in use such as CK and myoglobins. Myosin also has the advantage of being more sensitive and more stable in blood, since its maximum level is reached 48 h after the lesion and it remains in the blood for longer so that it is easier to use in diagnoses not carried out immediately and can be used to follow the development of the muscle lesion. We therefore conclude that the determination of fast and slow myosins is a useful aid in the diagnosis of muscle lesions, especially for those that are difficult to detect by other procedures. 
